# Patient experiences with person-centred and integrated chronic care, focusing on patients with low socioeconomic status: a qualitative study

**DOI:** 10.3399/BJGP.2024.0400

**Published:** 2025-05-20

**Authors:** Hester E van Bommel, Lena HA Raaijmakers, Maria ETC van den Muijsenbergh, Tjard R Schermer, Jako S Burgers, Tessa van Loenen, Erik WMA Bischoff

**Affiliations:** Radboud University Medical Center, Research Institute for Medical Innovation, Department of Primary and Community Care, Nijmegen and Pharos, Dutch Centre of Expertise on Health Disparities, Utrecht, the Netherlands; Radboud University Medical Center, Research Institute for Medical Innovation, Department of Primary and Community Care, Nijmegen, the Netherlands; Radboud University Medical Center, Research Institute for Medical Innovation, Department of Primary and Community Care, Nijmegen and European Forum for Primary Care, Utrecht, the Netherlands; Radboud University Medical Center, Research Institute for Medical Innovation, Department of Primary and Community Care, Nijmegen and Gelre Hospitals, Science Support Office, Apeldoorn, the Netherlands; Dutch College of General Practitioners, Utrecht and Department of Family Medicine, Care and Public Health Research Institute, Maastricht University, Maastricht, the Netherlands; Radboud University Medical Center, Research Institute for Medical Innovation, Department of Primary and Community Care, Nijmegen, the Netherlands; Erasmus MC, Radboud University Medical Center, Research Institute for Medical Innovation, Department of Primary and Community Care, Nijmegen and Department of General Practice, Erasmus MC, University Medical Center, Rotterdam, the Netherlands

**Keywords:** general practice, multimorbidity, low socioeconomic status, multiple chronic conditions, patient-centred care, primary health care

## Abstract

**Background:**

The effectiveness of single disease management programmes in general practice may be limited for patients with low socioeconomic status (SES), as these programmes insufficiently take into account the specific problems and needs of this population. A person-centred integrated care (PC-IC) approach focusing on individual patient’s needs and concerns could address these problems.

**Aim:**

To explore experiences of patients with (multiple) chronic diseases with regard to the acceptability of a general practice-based PC-IC approach, with a focus on patients with low SES, and to establish which modifications are needed to tailor the approach to this group.

**Design and setting:**

In 2021, a feasibility study in seven general practices in the Netherlands was carried out. The healthcare professionals provided care based on a PC-IC approach for patients with diabetes, chronic respiratory diseases and/or cardiovascular disorders. Patients were followed for 6 months.

**Method:**

This was a qualitative study using focus group discussions, in-depth interviews, and semi-structured telephone interviews in a total of 46 patients with chronic diseases and multimorbidity, including 31 patients with low SES.

**Results:**

An overall positive experience of participants with the PC-IC approach was observed. Discussing their health made patients feel they were being taken more seriously and seen as a unique individual, and it provided the opportunity to discuss their life and health concerns. Recommended adaptations of the PC-IC approach for patients with low SES include creating materials that are clear and easy to understand and offering communication training for healthcare professionals.

**Conclusion:**

The PC-IC approach seems helpful for patients with chronic diseases, provided that it is tailored to their skills and abilities. Several modifications for patients with low SES were suggested.

## Introduction

The increasing prevalence of chronic diseases has a huge impact on healthcare systems worldwide.^1^^,^^2^ In the Netherlands, more than 10 million patients (59%) have chronic diseases such as type 2 diabetes mellitus, chronic respiratory diseases, or cardiovascular disorders, and half of them have several chronic conditions (that is, multimorbidity).^3^ Patients with multimorbidity have a higher mortality rate, poorer health-related quality of life, higher healthcare costs, and more productivity loss compared with patients without multimorbidity.^4^ Patients with low socioeconomic status (SES), defined as education at basic or lower vocational level (26% of the Dutch adult population), two to four times more often have chronic diseases than patients with higher levels of education.^5^

In the Netherlands, the GP is the first contact for health-related issues and the gatekeeper in the healthcare system. Patients are obliged to register with a general practice and consulting a GP is free of charge, as the costs are covered by mandatory healthcare insurance. Evidence-based disease management programmes (DMPs) for patients with type 2 diabetes mellitus, chronic obstructive pulmonary disease (COPD) and increased cardiovascular risk have been introduced in the Dutch general practice to mitigate the impact,^6^ which are mainly delivered by practice nurses under supervision of a GP. As half of the patients had multimorbidity, the focus on one disease has limitations.^7^ Patients experience DMPs as fragmented, with limited attention to their personal context.^8^ Moreover, the effectiveness of the single DMPs may be lower in patients with low SES, as these programmes insufficiently take the specific problems and needs of this population into account.^9^

**How this fits in**

Effective chronic disease management is an essential part of general practice care. This is the first study, to the authors’ knowledge, on the experiences of patients using a new person-centred integrated care approach in the Netherlands, with special attention given to people with a lower socioeconomic status. The findings suggest the PC-IC approach is helpful for patients of any socioeconomic status, provided that it is tailored to their skills and abilities. It is important that healthcare providers are aware of the necessary adaptations for people with lower socioeconomic status when delivering chronic disease management.

A person-centred integrated care (PC-IC) approach focusing on individual patient’s needs, values, and concerns could address these problems.^10^^,^^11^ It puts the patient at the centre of care instead of the disease, and care is tailored to the individual patient needs. PC-IC approaches could reduce the impact and burden of chronic diseases and multimorbidity.^12^

The aim of our study was to explore experiences of patients with (multiple) chronic diseases with regard to the acceptability of a general practice-based PC-IC approach,^10^ with a focus on patients with low SES. In addition, we aimed to explore to what extent the procedures and materials used in this PC-IC approach are suitable for patients with low SES, and what would be needed to tailor the approach to the needs and skills of this particular group.

## Method

### Setting and design

From March 2021 to December 2021, a feasibility study was conducted in seven general practices which were situated in three different regions of the Netherlands. In each region we collaborated with the regional primary care cooperative: in the Nijmegen region the primary care cooperative comprised 168 GPs (approximately 290 000 registered patients), in the Arnhem region 193 GPs (approximately 440 000 registered patients), and in the Doetinchem region 116 GPs (150 000 registered patients). The primary care cooperatives, which are responsible for organising DMPs in general practices in their region, informed all practices about the study and called on them to volunteer to act as a pilot practice for the implementation of the previously developed PC-IC approach.^10^

Seven practices (Nijmegen region, three practices; Arnhem region, two practices; and Doetinchem region, two practices) volunteered to participate. The practice nurses and GPs in these practices provided care based on the PC-IC approach for patients with type 2 diabetes mellitus, chronic respiratory diseases, and/or cardiovascular disorders. Included patients were followed for 6 months. Quantitative data for this study will be published at a future date.

We assessed patient experiences of the PC-IC approach, using multiple qualitative methods. Owing to the constraints imposed by the COVID-19 pandemic, we conducted online focus group discussions (FGDs) with patients with varying levels of educational attainment, to explore their experiences with the PC-IC approach. Recognising the potential digital challenges faced by patients in participating in online focus groups, we chose to conduct additional individual interviews. Lower digital skills are more prevalent in people with lower SES. In this study, lower SES was defined as a lower educational level (having completed no more than post-secondary vocational education level 1, or equivalent) and/or having indicated a need for assistance in reading in the self-reported baseline measurement at the start of the feasibility study. After reaching data saturation in the interviews with low SES patients, the remaining participants with low SES from the feasibility study were invited for brief, structured individual telephone interviews to validate the findings. Data triangulation and researcher triangulation was therefore used to enhance trustworthiness of the data.

### Study population

Participants in the FGDs and individual interviews were purposefully recruited within the group of patients who were included in the feasibility study described above, aiming for diversity in terms of age, sex, educational level, migration background, geographical location, and GP. Recruitment continued until data saturation was reached.

Participants received information about the feasibility study before participation and gave informed consent. We adapted the information to ensure it was understandable for people with limited health literacy.

### The PC-IC approach

**Figure 1:**
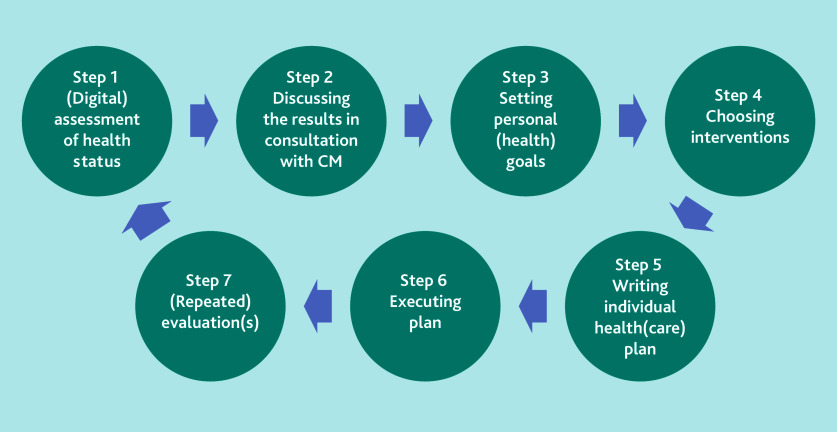
Schematic representation of the Person-Centred and Integrated Care (PC-IC) approach for the management of patients with chronic diseases and multimorbidity in general practice. Reproduced with permission from LHA Raaijmakers et al.^10^ Participating patients completed an integral assessment of their health status by discussing the different domains with the healthcare professional (HCP) (Step 1 and Step 2). In consultation with the HCP, the patient decided which domain(s) should have priority and defined the individual (health) goals (Step 3). Options for treatment or support are chosen that suit the patient (Step 4) and the goals and interventions selected are written in the digital individual care plan (Step 5) that can be shared with other HCPs involved. In Step 6, the selected interventions are provided. After or during the process one or more practice visits can be planned, depending on the patient’s needs (Step 7). After a predefined period, for example one year, the patient’s integral health status is reassessed and the process repeated. CM = case manager.

The PC-IC approach that was implemented in the feasibility study entails a systematic, cyclical process of seven steps that healthcare providers undertake in collaboration with patients, as illustrated in Figure 1.^10^ Key to this approach is a discussion (Steps 1 and 2) about all the patient’s concerns and needs, not just those related to the chronic disease(s), but also to the patient’s psychosocial wellbeing. To facilitate this discussion, there are different aids and tools available, such as the Positive Health Tool.^13^ This tool was often applied in the GP practices involved. It might help to focus the discussion on the patient themselves, on their resilience, and on what makes their life meaningful, encompassing the six domains of the Positive Health Tool: bodily functions, mental and emotional wellbeing, meaningfulness, quality of life, social wellbeing/participation, and daily functioning.^13^ Before the start of the feasibility study GPs and practice nurses followed training that equipped them with the knowledge and skills needed to integrate the new PC-IC approach into their consultations in practice.

**Figure 2:**
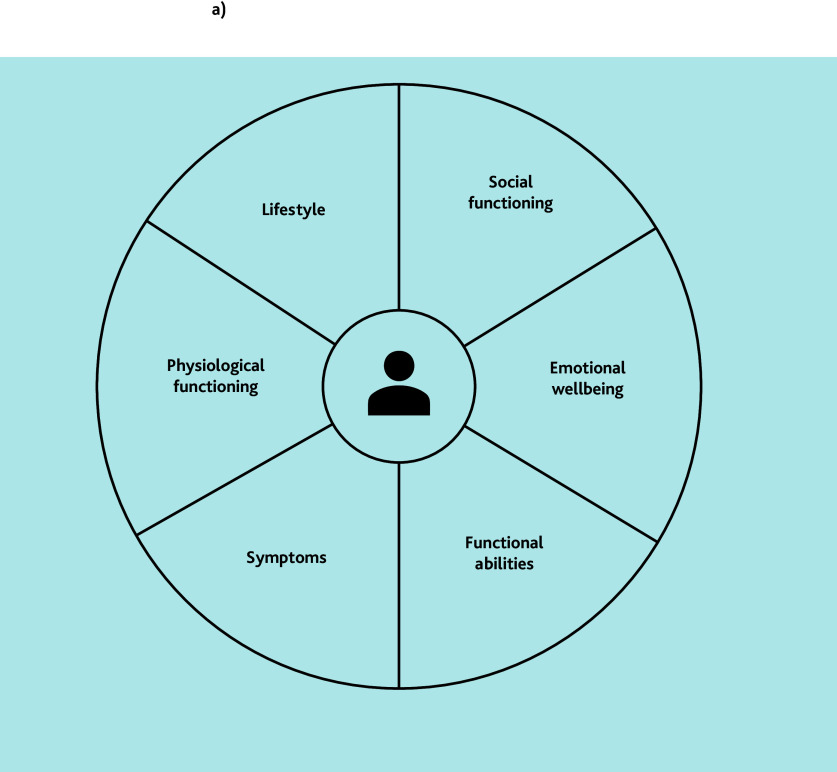
Domains of integral health status. The first step in PC-IC approach involves assessing the patient’s integral health across multiple domains, using a (digital) questionnaire at home combined with physical measurements.^10^ See Figure 1 for the detailed steps involved. Reproduced with permission from LHA Raaijmakers *et al*.^10^

### Data collection

#### FGDs

The FGDs were held online via a web videoconferencing platform (Zoom). The FGDs included five to eight individuals, with each session lasting between 60 and 90 min. An experienced facilitator led each focus group, with a researcher present to observe and assist with moderation as needed.

The FGD were guided using a topic guide (Supplementary Information S1) that was developed using the Consolidated Framework for Implementation Research.^14^ Main topics addressed the participants’ overall experiences with chronic care in general practice, their perceptions of changes introduced by the new PC-IC model, and their opinions on specific aspects of the model, such as the utilisation of surveys and visual aids.

#### Individual in-depth interviews

For the individual in-depth interviews the topic list for the FGDs was expanded (Supplementary Information S2) with items relating to the House of Care model^15^ and person-centred care literature.^16^ It included participants’ comprehension of questionnaires, experiences with the PC-IC approach, individualised needs, patient involvement and self-management, skills and attitudes of healthcare professionals (HCPs), the establishment of personal goals, and referral to other healthcare providers. These interviews were semi-structured, took 45–60 min each, and were conducted by several researchers.

#### Semi-structured telephone interviews

For the brief semi-structured telephone interviews, the topic list (Supplementary Information S3) was based on the same literature that was used for the individual in-depth interviews. It contained closed (*n* = 25) and open questions (*n* = 4) about the understanding of materials, feasibility of procedures, and experiences with the PC-IC approach. The telephone interviews took 20–30 min and were conducted by one researcher.

### Data analysis

The FGDs and in-depth interviews were audiotaped, transcribed ad verbatim, and then coded by several researchers independent from each other to ensure coding validity. For the FGDs, two researchers were responsible, and for the individual interviews three researchers were responsible for the coding. ATLAS.ti version 9.1.6) was utilised for this coding process. The results from the FGDs and the individual interviews were deductively coded according to the steps of the cyclical process of the PC-IC approach (Figure 1) in a framework analysis and were included in the coding tree. The responses of the semi-structured telephone interviews were recorded and also uploaded in ATLAS.ti.

Two researchers discussed the quotations and respective codes derived from the FGDs, and three researchers discussed those from the individual in-depth interviews and semi-structured telephone interviews, until consensus was achieved. We defined data saturation as finding repeating results within each category of the framework analysis.^17^ The study was reported according to the Standards for reporting qualitative research by O’Brien *et al*.^18^

All the researchers had received higher education, which might have influenced their perspective and experience with health care, and therefore the interpretation of the data. Three were trained in the medical field, which could positively influence their understanding of the medical experience of patients. Two researchers are experts in working with people with limited health skills, which was very helpful in understanding their experience.

## Results

### Characteristics of the participants

Ten patients participated in two FGDs, 11 patients in the individual in-depth interviews, and 25 patients in the semi-structured telephone interviews, resulting in a total of 46 participants. Out of these 46 participants, most participants (59% n = 27) were ≥65 years, and 63% (n = 30) of the participants had multimorbidity. In total, 31 of the 36 participants who participated in the individual interviews and telephone interviews had received primary or preparatory vocational education, which was defined as lower SES (Table 1).

**Table 1:** Characteristics of participants

Characteristic	Participants in focus group discussions, *n* (*N* = 10)	Participants in individual in-depth interviews, *n* (*N* = 11)	Participants in semi-structured telephone interviews, *n* (*N* = 25)
**Age**			
<65 years	5	6	8
≥65 years	5	5	17

**Sex**			
Male	6	4	6
Female	4	7	19

**Educational level**			
Primary education^a^	–	–	4^a^
Primary/preparatory vocational education^a^	–	8^a^	19^a^
Higher secondary education	1	–	–
Post-secondary vocational education	1	1	2
University of applied science	5	2	
University	3	–	

**Chronic diseases**			
COPD	–	–	1
Cardiovascular disease	4	3	8

**Multimorbidity (≥2 chronic diseases)**	6	8	16

a Lower socioeconomic status.

COPD = chronic obstructive pulmonary disease.

### Experiences with the PC-IC approach

Overall, the majority of the participants from the FGDs and individual interviews were positive about the PC-IC approach and with the consultation with their GP or practice nurse encompassing multiple domains. The participants felt very positive about being seen as a unique person, and not as someone with a disease, and appreciated the opportunity to talk more about their life and concerns.

Here, we discuss the results for each step of the PC-IC approach, focused on Steps 1 to 5. However, in addition to the new PC-IC model, participants also mentioned their own general care needs.

#### General care needs

Besides the new PC-IC approach, participants discussed their own needs concerning care and managing their chronic diseases. Most important to them was a good relationship and communication with their HCPs. They emphasised the significance of personal continuity of care, seeing the same HCP, and the importance of a case manager, whether a practice nurse or GP:

*‘But personally, I hardly know my GP. I met her once, and after that, when I go there again, there’s another substitute or so* […]*. To see the same person every time, I would, I would really love that.’* (FGD participant [P]7)

In addition, participants highlighted the need for understandable explanations and the opportunity to ask questions. They mentioned the need for sufficient time during consultations and particularly the perception of having time for a personal discussion and not being treated as someone with a disease:

*‘I think attention and being seen*. […] *That you feel like someone is listening to you. You can do that in 10 minutes. But if you feel comfortable, at ease, you are much more open to things.’* (FGD P8)

From the individual in-depth interviews it also emerged that short lines of communication between different HCPs was important:

*‘I think for anyone who needs care, it’s important and noticeable that there is good communication between them. And that you don’t have to tell your story everywhere. Short lines and not getting the feeling that you are a number, that has to go away.’* (In-depth interview [INT] P4)

#### PC-IC Step 1. Assessing integral health status

Participants in the focus groups indicated that the online Positive Health Tool to assess their integral health status was a good starting point for a structured consultation with their practice nurse or GP about their issues. The variety of domains in the questionnaire helped to focus more on social and psychological factors influencing their lives than on physical factors only. Completing the tool led to new insights for themselves and helped to set the agenda for the conversation with the HCP:

*‘I like it because it’s another snapshot. And that the outcome is discussed, because sometimes things come out that you fill in, that you don’t put the emphasis on, or that you don’t recognise. And via the spider web, you think, yes, I should pay more attention to that.’* (FGD P8)

In contrast, approximately half of the participants with low education found it difficult to complete the questionnaire digitally. Nineteen out of 36 participants from the in-depth interviews and semi-structured telephone interviews filled in the questionnaire digitally themselves, while 15 used a printed paper version instead. Two participants did not use the questionnaire.

*‘I prefer on paper. I cannot do it otherwise without help. I can handle it until the moment something is going wrong. It causes stress and on paper I can read it all back, which I find easier.’* (INT P3)

Ten participants needed assistance and filled out the questionnaire together with the practice nurse:

*Interviewer: ‘How did you feel about filling those out together?’*

*Participant: ‘That’s nice, because then you can talk about it and other things come up, then you get more out of it.’* (INT P7)

One of the practice nurses involved completed the questionnaires together with the participants, even with those who could read and write well, and participants appreciated this, as it gave them the opportunity to ask for clarification if the question was difficult to understand. One participant with low education mentioned that a questionnaire creates a lot of anxiety and stress if you cannot read well. It reduced a lot of stress if the practice nurse could help with this in advance.

#### PC-IC Step 2. Discussing health with HCP

Participants in both the FGD and individual interviews indicated that they valued discussing the outcomes about their integral health status with their HCP. Compared to before, participants felt they now had more opportunity to discuss their concerns about social aspects and daily life, or ask questions compared with when they received disease-oriented care. They also felt as though they were being seen more as a person rather than someone with a disease and were taken more seriously:

*‘The health care becomes a lot more concrete, and you have a lot of depth that way. It forces you to think more deeply, about your state.’* (Telephone interview [TI] P20)

Participants in the FGDs with multimorbidity mentioned that the connection between the individual diseases received more attention and led to new insights:

*‘Because we have probably figured out now, why I have seven chronic diseases. And by going through connections, and by looking at the whole picture for once, and that they can treat that, which might make my diseases either better or at least not worse. So for me this has just been a great outcome.’* (FGD P8)

Patients reported being more motivated for behavioural changes:

*‘The conversation is more on the person, which is positive, good to go a little deeper, and it helped to come to conclusions with the practice nurse.’* (TI P1)

Some participants with low SES had difficulties with understanding the ‘spider web’, which is a visual presentation of the results of the questionnaire about the six domains of positive health (Step 1). However, after clarification by the practice nurse they felt encouraged by the PC-IC approach to talk about personal issues. Discussing their integral health status made participants feel more positive about the relationship with the HCP:

*‘Now they take their time and things become more discussable and you tell them more quickly. So if I have a problem and people really ask me about it, I am more likely to say it.’* (INT P10)

#### PC-IC Step 3

##### Setting personal health goals

Some participants in the FGDs and individual interviews reported that preparing the session at home (Step 1 in the process of the new PC-IC approach) and discussing their integral health status (Step 2) improved their motivation to work on their personal (health) goals, and they felt more involved:

*‘Because we came up with the goals together, and I looked at home to see whether it worked out, I also had physical complaints and they disappeared because I got the right tools from the practice nurse to work on my health.’* (TI P10)

From the individual in-depth interviews and semi-structured telephone interviews, some participants reported collaborating with the practice nurse to identify the prioritised domain(s) and establish specific goals:

*‘Because it’s small simple things, we come up with ideas together and then I see if I can implement the goals. On my own I don’t come up with those ideas, and the conversation and the questionnaire helps me to break patterns. One of the goals, to be able to sleep better, has already succeeded because of that.’* (TI P7)

Eighteen of the 31 participants with low SES did not recognise themselves as having set personal goals, although they did receive some tips or advice to improve their health status. These participants with low SES mentioned that they get standard advice, which was not tailored to their needs or suitable for their (physical) condition.

Some of the participants with low SES reported that it was difficult to define their personal goals because of their daily life, or the practice nurse presented it too much as a ‘have to do’ instead of an option or advice:

*‘I like that my practice nurse knows my situation, now she knows why I find it hard to follow advice, it’s not that I don’t want to, but I’m too busy with other things and easily distracted.’* (INT P1)

#### PC-IC Step 4

##### Choosing treatment or support to achieve (health) goals

By discussing which treatment or support is suitable, most participants reported that they were more involved in decision making using the new PC-IC approach. However, other participants reported already being involved in decision making in usual care:

*‘You have much more interaction with each other than before. It was more one-way traffic in my case before* [the PC-IC approach]. *It was just this is what is happening and this is how we are going to do it and without consultation of how do you feel about it yourself and what can you do about it yourself, and things like that.’* (FGD P1)

Some participants with low SES experienced increased self-awareness and a deeper comprehension of the origins of their symptoms, facilitated by receiving suitable guidance on strategies to address them:

*‘Through the insight into your lab results, and also through conversation, a lot of things come up, the practice nurse addresses them, and therefore I know better what I can do.’* (TI P19)

#### PC-IC Step 5

##### Writing the personal healthcare plan

A majority of participants in the individual in-depth interviews and telephone interviews experienced confusion about the presence of a care plan because they were not aware of the presence of a personal plan in their electronic health record (EHR). Although accessible through their patient portal, participants, especially those with limited digital skills, rarely used it. Few received a concise care plan on paper. Some indicated that they preferred to receive it digitally, whereas others preferred to receive the information and appointments on paper, which they found easier to understand, aiding their memory of agreed-on goals and treatments at home:

*‘Prefer paper, I am not so into digital. I can grab it more easily ... but keep it understandable. I think some people need pictures to understand it and you can look it over again.’* (INT P4)

## DISCUSSION

### Summary

This study showed an overall positive experience for participants with this new PC-IC approach. Discussing their health made them feel they were being taken more seriously and seen as an individual, and it provided the opportunity to discuss all their concerns in daily life with their HCP. This positively influenced their motivation and ability to adhere to their treatment goals. However, the digital questionnaire on health induced stress for individuals with low literacy or limited digital skills. Moreover, the Positive Health Tool was deemed overly abstract and difficult to comprehend by the majority of participants with low education. These participants preferred to fill in the questions together with their practice nurse.

Important aspects of chronic care according to the participants are personal continuity of care, equitable conversations and understandable explanations from caregivers, the opportunity to ask questions, and the feeling of being given enough time.

### Strengths and limitations

A strength of the study was that most of the patients included had lower levels of education. We adapted the informed consent information for participants so it would be appropriate for a wider range of patients and organised individual telephone interviews with them, resulting in a high participation rate.

One of the limitations is the shift to online FGD necessitated by COVID-19 measures, which posed challenges, especially for participants who lacked digital skills or internet access. However, conducting supplementary individual in-depth interviews and brief telephone interviews with participants with low SES or participants with limited literate skills provided ample insight about this subgroup.

A second limitation might be the generalisability of our study findings, owing to the participation of three regions relatively close together in the Eastern part of the Netherlands. However, the seven participating practices in three regions serve a mix of rural and urban populations including groups with social deprivation and ethnic diversity. Therefore they may be considered a fair representation of the Dutch general practice population.

Selection bias is another potential concern because possibly only patients who were enthusiastic about the new PC-IC-based care that they had received wanted to participate in the focus group and individual interviews. This probably also applies to those participating in the entire feasibility study, in which the interviews were embedded. The majority of participants were native-born Dutch. Although patients with a migration background (that is, born outside the Netherlands, or second generation migrants) were part of the population served by the participating practices, few took part in the study.

Another limitation may be that in research on the relationship between SES and health, the indicators, education, income, and occupation are often used. We chose to use only education level (highest completed education) as a measure of SES, rather than also including income and occupation. Education level is a common measure of socioeconomic health disparities.^19^ The level of education determines the access to information and the ability to use new information (and thus health literacy), and it is an important determinant of income and occupation.^20^ In this study, we focused solely on education level to classify participants into socioeconomic status categories. As a potential improvement for future research, employment status could be considered to further refine the SES measure. To evaluate health literacy, we included a question from the Single Item Literacy Screener.^21^ While we acknowledge that individuals with lower levels of education can possess strong literacy skills — and conversely, those with higher education may have limited skills — such instances are relatively rare.

This study was limited by the absence of participants with language barriers who are often underrepresented in research. However, we could not include participants without Dutch language skills or community support. Unfortunately, we have no data on participants’ first-language preferences.

Although this feasibility study included patients with low SES, all researchers involved were highly educated. This may have introduced a bias in the interpretation of the results, given the differences in living context between the researchers and the participants.^22^

### Comparison with existing literature

Other countries are also implementing PC-IC approaches, such as the UK with the 3D approach for patients with multimorbidity in general practices.^23^ To our knowledge, they have not published a qualitative study of patient experiences with their approach and we have not found other qualitative research on patient experiences of PC-IC approaches within the context of general practice. However, other elements of our results, such as the importance of personal continuity of care, is well-described in previous research.^24^^,^^25^

In addition, the results of our study are consistent with findings from previous studies that showed that, to increase patient satisfaction, HCPs should communicate effectively, using clear, concise language, being empathetic, asking the patient whether they understand everything, and paying close personal attention.^16^ By doing this, the patient will be more involved in decisions made about their care and treatment, which could lead to better treatment planning and compliance.^26^ A therapeutic alliance and better health (system) results depend on the HCP’s respectful and sympathetic tone of voice. In addition, creating a personalised care plan in collaboration with all relevant stakeholders can have a favourable impact on the patient’s ability to manage their own health.^27^ However, in our study, the impact on patients in the low education group was not conclusive, as we did not determine whether the care plan effectively influenced their ability to manage their health as assessing this was beyond the scope of the current research.

However, as some patients with limited digital skills in our study had difficulty accessing their EHR and therefore could not read their personal healthcare plan, attention should be paid to making this plan more accessible and easy to read. It would be beneficial for all HCPs involved as well as the patient to share an electronic record of the healthcare plan, which could include a means of collaboration. For example, (welfare) professionals in other domains like social services, cannot see a digital record by the GP or practice nurse currently.^28^ Health information exchange could enable secure, real-time sharing of patient information across various locations, reducing redundant tests, and improving care quality by facilitating access to EHRs. However, the adoption of EHRs can present challenges, such as financial costs, workflow disruptions, privacy concerns, and other unintended consequences.^29^ For patients with limited health skills, potential benefits might be better coordinated care, because of better information exchange, but risks include unnecessary sharing of sensitive information with care providers. In some regions in the Netherlands, systems are implemented for collaboration between patients, informal care providers, and HCPs, in which data can be shared securely and patients have control over which party gets access to specific information.^30^

### Implications for research and practice

To make the new PC-IC approach suitable for all patients, easy to understand materials should be developed in co-creation with patients with limited literacy. Questionnaires to assess health status should be available on paper and not only online. Practice nurses should offer to complete these questionnaires together with the patient. Treatment plans should be drawn up for all patients with chronic diseases or multimorbidity, according to their own goals and should be accessible for the patient. This includes ensuring that information in the patient record is presented in a way that is clear and easy to understand.

Preferably, general practices should offer personal continuity of care and HCPs receive training to communicate effectively by using simple language, emphasising points, using teach-back methods, and listening intently.

Further steps are currently being taken to evaluate the PC-IC approach in which care is tailored to the individual patient with chronic conditions and multimorbidity on relevant outcomes such as patient experiences and population health in a cluster randomised-controlled trial. It is important to include as many different patients perspectives as possible, which includes understudied groups such as ethnic minorities, patients with literacy issues, and patients with low levels of education. Therefore, we are paying special attention to these groups in an additional cluster randomised-controlled trial.

In conclusion, the new PC-IC approach is helpful for all patients with chronic conditions provided that it is tailored to their skills and abilities. Adaptations of the PC-IC approach to patients with low SES include developing materials that are easy to understand, tools to recognise patients with low health literacy or complex social problems, offering communication training to HCPs, and adjusting the language accordingly.

## Supplementary Information


