# Supplementary material for: Patient experiences with person-centred and integrated chronic care, focusing on patients with low socioeconomic status: a qualitative study
Source: Br J Gen Pract. 2025 May 20;75(755):e423–30. doi: 10.3399/BJGP.2024.0400 (PMC12117502; doi:10.3399/BJGP.2024.0400)
Supplement: Supplementary file 1 [file BJGP.2024.0400_suppl.pdf]

Supplementary Information S1: Topic guide focus groups

- Starting questions
  - What differences did you notice between usual care and the PC-IC approach?
  - What are your experiences with the PC-IC-approach?
  - What would you like to change about the approach?

Topic		Questions
I. Intervention characteristics	A. Intervention source	
	B. Evidence strength & quality	
	C. Relative advantage	How does the PC-IC-approach compare with usual care in general practice?<br>1. What advantages does the "new" way of care have compared to the care you received before? Which components contributed most to this?<br>2. What disadvantages does the 'new' way of care have compared to the care you received before? Which components contributed the most to this?
	D. Adaptability	What should be changed to make the new care the best it can be? Why?<br><br>What should not be changed to keep the new kind of care as good as possible? Why?
		How do patients experience the expertise of healthcare providers?
	E. Trialability	How did patients experience the questionnaires?<br>Points of improvement for evaluation study?
	F. Complexity	How complex is the intervention? What makes it complex? What would help make it less complex?
	G. Design quality	What did you think of the support resources available, such as online resources or visual aids? What materials are still needed?
	H. Cost	
II. Outer Setting	A. Patient Needs & Resources	In what ways does the new care program meet the needs of patients?<br><br>What are the barriers to patients receiving the new care?
		In what way were you helped to achieve your goals?
	B. Cosmopolitanism	
	C. Peer Pressure	
	D. External Policy & Incentives	
III. Inner setting	A. Structural Characteristics	
	B. Networks & Communications	What does the patient notice about mutual cooperation between different healthcare providers? What would you like to see different?

	C. Culture	
	D. Implementation climate	
	E. Readiness for implementation	
IV. Characteristics of individuals	A. Knowledge & Beliefs	What feeling does performing this intervention evoke in patients?
	B. Self-efficacy	
	C. Individual Stage of Change	
	D. Individual Identification	
	E. Other Personal Attributes	
V. Process	A. Planning	
	B. Engaging	
	C. Executing	
	D. Reflecting & evaluating	

Supplementary Information S2: Topic guide in depth interview patients

General

1. How did you like participating in the new care program? What was good, nice, not nice or not good about this? What grade would you give the new care program on a scale of 1-10? Why?

Questionnaire

2. You were given a questionnaire to complete in advance. How did you find completing the questionnaire? Score of 1-10? What would it take to get this score up?

   a. Did you do this digitally or on paper?

   b. Did you complete the questionnaires alone or with someone (and why together?)?

   c. What did you think of the questions? Were they difficult or easy? A lot or just right?

   d. Many people often find questionnaires difficult to fill out, how is this for you?

      i. What should the questionnaire look like so that you find it easier to fill it out?

3. Did you feel, by completing the questionnaire, that you thought more about your illness/complaint/affliction, than before/before? What caused this, or why not?

Discuss results

4. After completing the questionnaire, the PN or GP [name} went to discuss it with you.

   a. What did you think of this? Score of 1-10? What would it take to get this score up?

   b. How did the discussion go (understandability)?

   c. Did the PN / GP also use a picture with it? Were you also given this to take home, or would you have liked it and why? (assume a picture/visual such as spider web)

5. What was it like for you to discuss the results like this after? Did you have a different conversation than before, i.e. before you participated in the study? What was different? How do you feel about that?

6. How do you feel about using pictures or images in the conversation with the PN /GP [name]? (In face to face interview, I would also like to show different conversation cards here, with pictures and topics).

7. Is there anything else that would help you or what wishes you have when discussing the results? (think: images, read-aloud, leaflet, more frequent contact)

Broad context patient

8. The PN / GP asks more than just about your illness, she also asks questions about e.g. your home situation or about your work. What do you think about that? Score of 1-10? What would it take to get this score up?

   a. Do you think it is important that the PN /GP [name] also knows more about your life? Why yes/no?

   b. Is this different from the care you received before this research/study?

Goals/ deciding together

9. Were (treatment) goals made (drawn up)? What did you think of them? Score of 1-10? What would it take to get this score up?

   a. Who made these goals? What role did you yourself have in making these goals?

   b. Where were these goals written down? Or can you view/see these goals anywhere?

   c. Were you given these goals to take home, would you?

   d. Did setting up the goals also give you confidence to achieve them? why and why not?

   e. What is needed to increase confidence?

10. Do you now know more or better what you can do yourself? If so what is that exactly?

    (e.g. following advice, complications, medication intake (understanding disease))

11. What other advice were you given, or were you referred (referred) to another healthcare/help provider? If yes, to whom? Is this different from before?

12. Were you also given information on paper or online to help you achieve your goals? What information? How do you feel about this information (If not, what kind of information would you need?)?

13. In what other ways did the healthcare provider help you achieve your goals? (Support)

Wishes/needs

14. What would you like in the care you receive, what is important to you? why?

15. What do you currently miss in the care or treatment? Information about illness, referral, communication

16. What are you very satisfied with now in the care or treatment? Why?

17. What are you less satisfied with in care or treatment now? Why?

18. What wishes do you still have for the care you receive? Why?

Caregiver

19. How is the contact with your caregiver? What grade would you give for this? Why? How could this be improved?

20. Do you feel that the PN / GP understands you well? why/why not?

21. Does the PN / GP [name] explain to you clearly, why/why not? are a lot of difficult words used?

22. Do you feel you can ask and tell anything to the PN / GP, why?

23. Is there a healthcare provider you can go to first with all your questions? Or to clarify: Or are there multiple caregivers you have to go to with your different questions?

24. Are there any other things you would like to discuss?

General questions

Gender: [need not be asked explicitly].

Profession:

Education completed:

Age:

Number of years in care with PN/GP and for what:

Where were you born: (for participants with a migration background also ask how long have you been in the Netherlands):

Supplementary Information S3: Topic guide semi-structured telephone interviews

General

1. How satisfied are you with the new care program?

   What grade do you give the new care program on a scale of 1-10?

   (1 is very poor, 10 is very good)

2. Has your experience of the care changed compared to before?
   - o Yes
   - o No
   - o I don't know

Questionnaire

3. How did you fill out the questionnaire?
   - o On paper
   - o Digitally, on a computer
4. Did you complete the questionnaire by yourself or together with someone else?
   - o Alone
   - o Together
5. What did you think of the questionnaire you had to complete, on a scale from 1 to 10?
6. Were the questions understandable?
   - o Very understandable
   - o I had difficulty understanding
   - o I don't know

After completing the questionnaire, you discussed the results from the questionnaire with your PN or GP [name]. This is what the following questions are about.

7. How did you feel about discussing the questionnaire, what grade do you give it?

   1 = I think it is not important or not necessary, 10 = I think that is very good, it is very important.

   Why...........? What do you think is good? What is needed to make the grade higher?

8. How understandable was it for you to discuss the results after?
   - o Very understandable
   - o I found it difficult to understand
   - o I don't know
9. Was a picture used when discussing the results? (Think spider web, discussion map)
   - o Yes: what grade do you give this? 10 = very good and clear 1= is not necessary for me
   - o No: would this have helped you? Yes/No
   - o I don't know

Broad context

10. The PN / GP [name] sometimes asks more than just about your illness, she also asks questions about e.g. your home situation or about your work. What grade do you give? ....

(10 = I think that is very good, 1 = I don't think that is necessary, 5 = I don't care).

11. Did you discuss your personal situation with the PN /GP before? That is, before you participated in this study/research?
    - o Yes
    - o No
    - o Somewhat

Goals/ deciding together

12. Do you discuss with your PN / GP how you want to work on your health?
    - o Yes
    - o Sometimes
    - o No

If no: Why don't you discuss how you want to work on your health? (You can give multiple answers)

- o I would like to discuss this, but my healthcare provider does not give me space to do so
- o I don't know what I want to work on
- o I would rather discuss this with another healthcare provider
- o I don't have enough information to do this
- o I do not feel the need to do so
- o It is new to me that I can discuss this with my healthcare provider.
- o Something else: ....

13. Have any (treatment) goals been established?
    - o Yes
    - o No
    - o I don't know
14. How do you rate that goals are/are not established? What grade do you give? 10 = I think it is very good, 1 = I don't think it is necessary, 5 = I don't care.
15. Who made the (treatment) goals?
    - o PN /GP
    - o Myself
    - o Together
    - o I don't know
16. What do you think of the treatment goals, do they fit you and your (personal) situation well?
    - o Yes, these goals fit me
    - o No, these goals do not fit me
    - o Somewhat

- Why do they fit (or not fit)? ....

17. Can you read or look up these goals anywhere?
    - Yes, I have them on paper
    - Yes, I can find them in my digital file/care plan
    - No, I do not know where I can find the goals
    - No, I cannot find them digitally (file/care plan)
18. Have you been referred by the PN to the community, another healthcare provider or course? E.g. to a dietician or physiotherapist or an activity on exercise or something like that?
    - Yes, but this was before I participated in the new care program
    - Yes, to achieve the goals I came up with in the new care program with the PN.
    - No
19. Because of the new care program, do you now know better what you can do yourself?

    (think about : following advice, complications, medication intake (understanding disease), more confidence)

    o Yes

    o No

    o Don't know

    Why yes/no......................

Satisfaction

20. Can you ask your healthcare provider the questions you want to know?

    o Yes

    o Sometimes

    o No

    If no: Why don't you ask your healthcare provider questions? (You can click more answers)

    o There is no time to ask questions

    o I don't feel comfortable asking questions

    o My healthcare provider does not know enough about my illness

    o My caregiver does not listen to me well

    o Something else: ....
21. Name one thing that would make the (new) care program even better?

General questions

- male
- Female

What is your age:.................

Education completed:

- Elementary school/ primary education
- Preparatory secondary vocational education
- The first three years of higher general secondary education / university preparatory education
- Post-secondary vocational education (level 1)
- Higher general secondary education / university preparatory education
- Post-secondary vocational education (level 2),
- Post-secondary vocational education (level 3)
- Post-secondary vocational education (level 4)
- University of applied science
- University

Profession:

Where were you born?.....................

Where were your parents born?............
